# Binding lies

**DOI:** 10.3389/fpsyg.2015.01566

**Published:** 2015-10-14

**Authors:** Avraham Merzel, Ilana Ritov, Yaakov Kareev, Judith Avrahami

**Affiliations:** ^1^School of Education, The Hebrew University of JerusalemJerusalem, Israel; ^2^The Federmann Center for the Study of Rationality, The Hebrew University of JerusalemJerusalem, Israel

**Keywords:** lies, binding, motivation, profit, commitment, self-presentation, impression management

## Abstract

Do we feel bound by our own misrepresentations? Does one act of cheating compel the cheater to make subsequent choices that maintain the false image even at a cost? To answer these questions we employed a two-task paradigm such that in the first task the participants could benefit from false reporting of private observations whereas in the second they could benefit from making a prediction in line with their actual, rather than their previously reported observations. Thus, for those participants who inflated their report during the first task, sticking with that report for the second task was likely to lead to a loss, whereas deviating from it would imply that they had lied. Data from three experiments (total *N* = 116) indicate that, having lied, participants were ready to suffer future loss rather than admit, even if implicitly, that they had lied.

## Introduction

There are many reasons why people lie: to obtain material benefits, to impress, to save themselves from embarrassment or inconvenience, to avoid punishment, to protect a relationship, or even to benefit others (through white lies) (Hample, 1980; Camden et al., 1984; DePaulo et al., 1996; DePaulo and Kashy, 1998; Robinson et al., 1998; Vrij, 2000).

Although often beneficial, lies also bear some costs: lies violate the actual or perceived consistency, which is one of the foundations of interpersonal relationships (Cialdini et al., 1995). Lies degrade the quality of the information conveyed, thus diminishing the ability to arrive at an informed, high-quality decision (Lewicki, 1983). Lies impair interpersonal communication (Lewicki, 1983; Millar and Tesser, 1988; Grice, 1989). Lying entails internal psychological costs to the liar (Mazar and Ariely, 2006; Mazar et al., 2008). Finally, getting caught lying arouses negative emotions that affect both sides (Lewicki, 1983; Sagarin et al., 1998), and may result in actions (like punishment) against the liar (e.g., Lewicki, 1983; Mazar et al., 2008). It is thus obvious that people would be more likely to lie when they are not afraid of being exposed (Schlenker, 1975; Baumeister and Jones, 1978; Silverman et al., 1979; Baumeister, 1982; Leary and Kowalski, 1990; Mazar et al., 2008).

It is often the case that the behavior that follows lying may determine the likelihood of the lie being detected. It is therefore plausible that people would choose to act in a way that minimizes the chance of being exposed. But to what extent? Would people be ready to forgo a benefit in order that a future action does not reveal that they had previously lied?

In the present study, we examined whether, and to what extent, a person who lied is committed to the lie. Specifically, we wished to see if future actions made by that person would be affected by the commitment even at the cost of forgoing some profit.

We are not the first to study behavior that follows dishonest acts. For example, Both Mazar et al. (2008) and Chance et al. (2011) gave participants tasks that assessed their ability, while having the opportunity to dishonestly inflate their performance. Following these tests participants were asked to predict their future performance in a similar task, this time without the opportunity to lie (for predictions of their past performance see Mazar and Hawkins, 2015). Their payment was determined both by their performance and by the accuracy of their prediction. Participants over-estimated their future performance. The authors interpret this over-estimation as reflecting self-deception. We wish to consider an alternative interpretation: impression management.

The study of lying behavior suffers from an inherent difficulty: on the one hand, to identify an action as a lie, one needs to observe actual behavior and compare it to the true state of affairs which is known, both by the potential liar and by the researcher. On the other hand, if participants are aware of being watched (i.e., that their lies could be exposed), the probability of them lying decreases (Mazar and Ariely, 2006).

One of the ways to deal with this dilemma is to set up a situation in which it is clear that no single lie can be caught. At the same time, liars can be identified with high probability by the degree to which the aggregate of their single, unverifiable, reports deviates from some reference value. Such a reference point can be based on the behavior of other people who could not lie when performing a similar task (Mazar and Ariely, 2006; Mazar et al., 2008; Chance et al., 2011; Schurr et al., 2012) or on statistical probability (Shalvi et al., 2011; Fischbacher and Föllmi-Heusi, 2013; Hilbig and Hessler, 2013). In the present set of studies, we employed a paradigm of the latter type: the participants repeatedly performed a task in which they could falsely report a favorable outcome, with no fear of being caught. However, a comparison between the proportion of trials in which a favorable outcome was reported and the expected proportion of such outcomes could indicate whether the participants were likely to have lied. A subsequent task then tested how committed the participants were to their lies (if they lied). We describe our paradigm in the next section.

## The Experimental Paradigm

The paradigm used here consisted of two tasks: a reporting task and a prediction task. In the reporting task the participants drew, privately, marbles from an opaque urn. The urn contained 100 marbles of two colors, and on each of 40 rounds the participants drew a marble and reported its color. One of the two colors was designated as the valuable color and reporting this color was rewarded. Thus in this task the participants had a financial incentive to inflate the number of marbles of the rewarding color, and did not run the risk of being caught lying. In the second task, which had not been announced in advance, the participants were asked to predict the number of marbles of the rewarding color that would show up in a sample of 40 marbles. Reward for performance in this task was based on the accuracy of the prediction. Obviously, the number that would most likely be accurate would be the number actually observed during the reporting task, rather than the number reported – if the latter had been inflated to obtain a larger reward. However, predicting a number that largely deviated from the value previously reported would constitute an admission, even if only tacit and indirect, that one had previously lied. Importantly, in our studies such a deviation would have no financial cost (the opposite: it would be financially rewarding). The only immaterial consequence would be that one would indirectly expose their lie. The question we explored was whether, and to what extent, participants whose reporting indicated they had most likely lied would be willing to suffer an expected loss, by predicting a value that was congruent with their previous report.

In what follows, we report three experiments that used this paradigm. Experiment 1 involved two phases, each consisting of a reporting and a predicting task, but with the second reporting task not incentivized. Experiment 2 used a preliminary, unincentivized, reporting task to rule out an alternative explanation based on anchoring. In Experiment 3 we introduced a procedure that guaranteed that reporting was anonymous to see if, when one’s prediction could not be associated with one’s previous reporting, participants would still be committed to their lies.

The experimental method employed in all three experiments reported was approved by the Human Subjects Committee of the School of Education, The Hebrew University of Jerusalem. All the participants signed an informed consent form before taking part in the experiments.

## Experiment 1

The purpose of this experiment was to demonstrate that people are willing to forgo a possible profit in order to keep the false representation they displayed. It was further designed to address an alternative explanation based on the misperception of small probabilities.

### Method

The experiment consisted of two, within-subject phases, each calling for the performance of the two tasks of reporting and predicting described above. The tasks of the second phase were identical to those of the first phase save for the fact that there was no incentive to lie during the reporting task. With, presumably, no lying in the latter reporting task the value in the prediction task was expected to correspond to the true proportion of marbles in the urn.

In the first phase the urn contained 35 green marbles and 65 yellow marbles and in the second phase it contained 35 blue marbles and 65 white marbles. The participants were informed that there were 100 marbles in the urns but they were not informed either of the number of marbles of each color or of the colors’ ratio. In the reporting task, a laptop computer was located next to the urn. Both urn and computer were hidden from the experimenter by a curtain and were visible only to the participant. There were two keys on the computer screen, corresponding to the colors of the marbles in the urn. The participant was to draw a marble from the urn and report its color by clicking the corresponding key on the computer screen. The marble then had to be put back in the urn and the urn shuﬄed. The participants were instructed to repeat this procedure 40 times. Importantly, at that point the computer stopped and displayed to the participant the number of the less common color (green in the incentivized reporting phase and blue in the unincentivized reporting phase), out of 40, that he or she had reported. At this stage the experimenter pulled the curtain aside, so the urn was visible to both the participant and the experimenter, and the predicting task began. The participant had to predict the number of marbles of the less common color in a sample of 40 marbles, drawn from the urn. After the prediction had been made and noted, the participant drew the marbles one at a time, and placed each marble in one of two separate containers, sorting the marbles by their color. Once 40 marbles had been drawn the experimenter and the participant counted the number of marbles of the relevant color together and compared it to the participant’s prediction.

In the incentivized reporting task, every time the participant pressed the “green” key, the computer added 0.5 NIS (New Israeli Shekels, 1 NIS being worth 0.26 $ at the time of the experiment) to the participant’s profits. It should be noted that the participants could report any color they wished without being exposed. The procedure of reporting in the second reporting phase was identical, but did not produce profit. This latter phase was introduced to make sure that the participants could correctly report the number of marbles of the infrequent color after 40 draws when there was no monetary incentive to inflate that number.

In the predicting task of both phases, payment was for *accuracy* in predicting the number of marbles of the infrequent color. The payment for a perfectly accurate prediction was an additional 5 NIS, and for a prediction that deviated by one from the number of marbles actually drawn it was 2 NIS. A prediction that deviated by more than one was not rewarded. Note that in the first phase this payment schedule could pose a dilemma for the participants who lied in the reporting task. On the one hand, their best prediction would have been the number of green marbles that they had *actually* drawn; on the other hand, if they were bound by their lies (i.e., lied and didn’t want to get caught lying) they should predict the number of green marbles that they *reported* in the previous task. Clearly, in the latter case, those who inflated the number of green marbles could expect to forgo the reward for accuracy of prediction.

### Participants

Thirty four students of the Hebrew University of Jerusalem were recruited for the experiment. The average age was 25.36 years (*SD* = 3.42 years) one student was excluded from the analysis, because he admitted that he didn’t understand what was required of him. 14 of the 33 participants were females.

### Procedure

Participants were tested individually in an empty classroom. The experimenter confirmed that the student could distinguish between the different colors of the marbles; those who had difficulty doing so were dismissed.

The instructions of the incentivized reporting task were read aloud and explained; written instructions were also available on paper in front of the participant. The same sequence of reading aloud and explaining, as well as providing written instructions, was true for all tasks.

After the instructions were explained, the participant went behind the curtain and started the task. Once the reporting task was over the prediction task started, with the participant predicting the number of green marbles that would be drawn out of 40 marbles. After that, the participant drew 40 marbles from the urn and sorted them by color into two boxes. The experimenter and the participant then counted the green marbles and the result was recorded. The second phase followed, starting with the reporting task and continuing with the prediction task for the blue/white urn. Finally, the total amount earned was calculated and paid, and the participant was dismissed.

### Results

**FIGURE 1:**
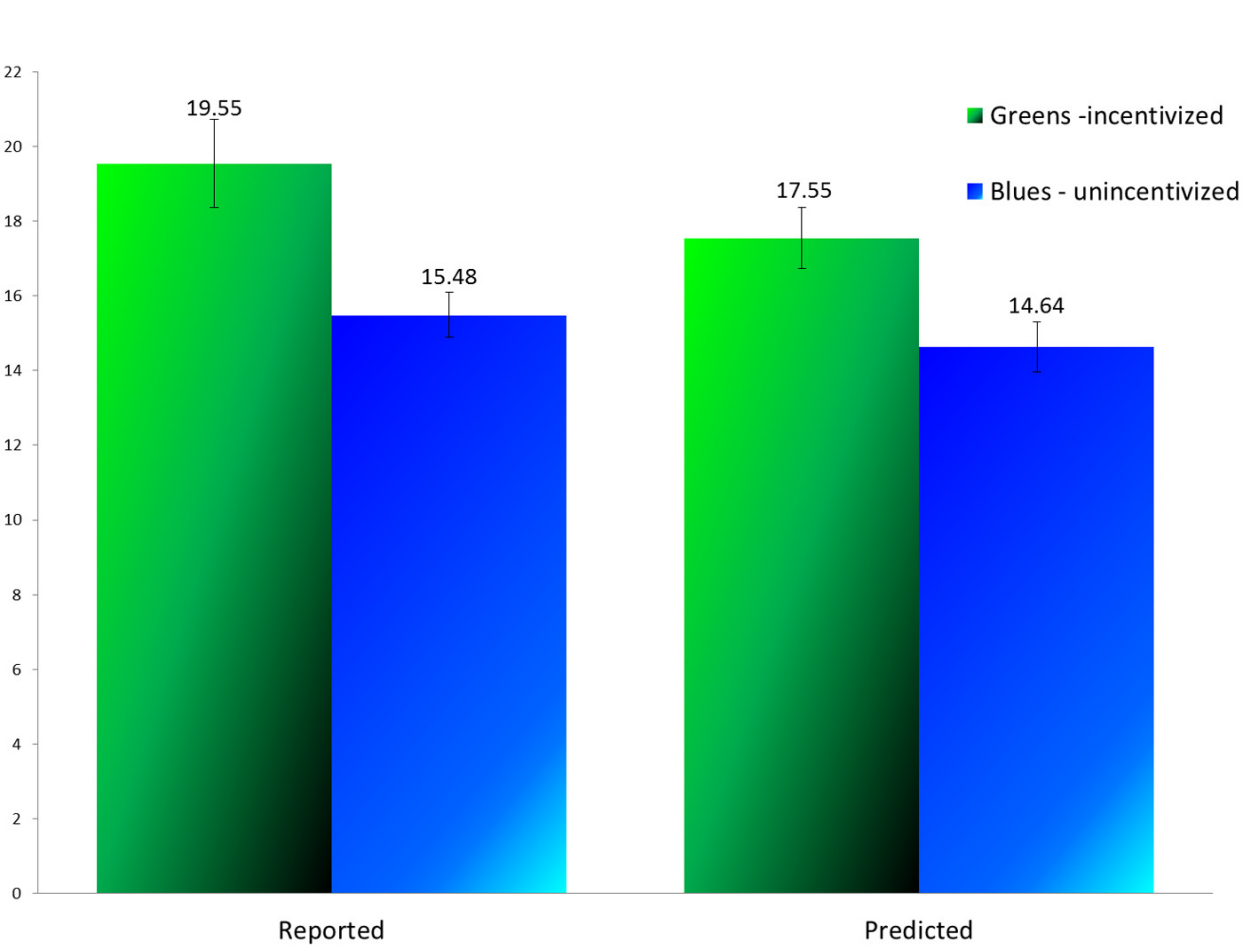
**Average of non-frequent marbles (reported or predicted) by phase (Experiment 1)**.

The mean number of the infrequent color, reported and predicted, for each phase and task, is presented in **Figure 1**. A two-way repeated measures ANOVA was conducted to test for the effects of Phase, Task, and their interaction. We found significant main effects of Phase, with the mean number higher in the first than in the second phase [*F*(1,32) = 10.583, *MSE* = 37.867, *p* = 0.003, $\eta_{p}^{2}$ = 0.249], and of Task, with the mean number higher in the reporting than in the predicting task [*F*(1,32) = 6.145, *MSE* = 10.893, *p* = 0.019, $\eta_{p}^{2}$ = 0.161]. The interaction was not significant [*F*(1,32) = 0.950, *MSE* = 11.518, *p* = 0.337, $\eta_{p}^{2}$ = 0.029).

While the results presented above capture the overall picture, it is easier to answer our research questions by reporting the results of pre-planned contrasts. First, the number of rewarding marbles reported in the first reporting task, in which misreporting was incentivized, was 19.55 – a value much higher than the value of 14, expected by chance [*t*(32) = 4.67, *p* < 0.001]. The prediction made following the incentivized reporting task, at 17.55, was also significantly larger than 14 [*t*(32) = 4.36, *p* < 0.001], indicating that the participants, although predicting a value somewhat lower than the one they had reported, were still committed to their lies. In fact, significant differences were observed not only between the reporting in the incentivized and unincentivized tasks [*t*(32) = 3.070, *p* = 0.004], but also, and most importantly, between the predictions made [*t*(32) = 2.609, *p* = 0.014]. The latter result indicates that the participants were ready to incur a loss in the predicting task to avoid having their prediction expose their lie.

As expected, the prediction following the unincentivized reporting task of the second phase (14.64) was not significantly higher than 14. However, the report in that phase (15.48) was, in fact, higher [*t*(32) = 2.488, *p* = 0.018]. We have no explanation for this result but it may be another indication of binding lies: participants may have suspected that the second reporting task could somehow expose their having lied before. Either way, the value of 15.48 is significantly different from either 19.55 [*p* = 0.004) or 17.55 (*p* = 0.032) in the incentivized phase.

To check if these findings may have resulted from a misperception of a difference in the statistical properties of the two procedures, reporting (calculating the expected proportion when drawing from an urn with replacement**)** and predicting (calculating the expected proportion when drawing from an urn without replacement), we ran a control study with 39 subjects, none of whom had participated in the other study. In this study participants were presented with a written description of the procedure and results of the previously ran study, and asked what could have brought about these results. Only one of the 39 subjects gave an answer that could have been interpreted as referring to a difference between the statistical probabilities in the two procedures.

**FIGURE 2:**
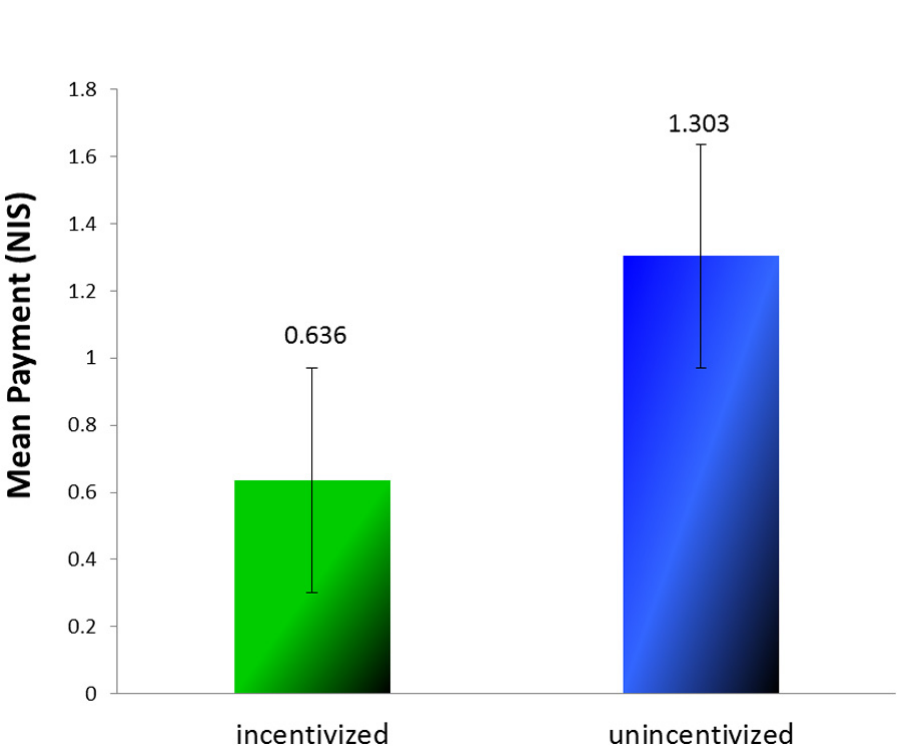
**Mean payment for prediction in the two phases (Experiment 1)**.

A comparison of earnings in the prediction tasks of the two phases (see **Figure 2**) revealed that earnings in the first phase (Mean Payment = 0.636 NIS, *SD* = 1.517) were indeed lower than that in the second phase [Mean Payment = 1.303 NIS, *SD* = 1.811; *t*(32) = 1.785, *p* = 0.042, one tailed]. In other words, had participants predicted what they must have *actually* observed while performing the incentivized reporting task they could have earned twice as much than they did. All in all, we conclude that the participants were willing to risk future gains rather than tacitly admit having lied before.

## Experiment 2

It could be argued that the inflated number of green marbles reported (and then displayed) in the incentivized reporting task did not bind participants but served as an anchor for the next task. In other words, this claim means that the participants knew that while performing the reporting task they had retrieved fewer green marbles than they reported. It is therefore possible that, when they had to predict the number of green marbles that would be drawn; their correct estimate was drawn upward through anchoring, which resulted in an intermediate value.

In Experiment 2 we addressed the anchoring issue, while replicating the previous results.

### Method

Experiment 2 was similar to Experiment 1, but included a preliminary, unincentivized reporting task, and no second phase. In the preliminary reporting task the participants did exactly what they did later in the incentivized reporting task, but they did not get any money for reporting “green.” For the preliminary task to make sense we asked the participants to use tongs to pull the marbles out of the urn – which was no simple matter –the unincentivized reporting task was described as “practice.” At the end of the practice task the number of green marbles participants reported was presented to them on the computer screen. Following practice the participants performed the second, incentivized, reporting task followed by the prediction task – both identical to the tasks performed in Experiment 1 (except for the requirement to use tongs to draw marbles one by one). As before, in both reporting tasks it was possible for the participants to report that they had drawn a green marble even if it was of the other color. In this experiment the practice task served as a control in that it provided another possible anchor.

### Participants

Thirty nine students of the Hebrew University of Jerusalem were recruited for the experiment. The average age was 25.64 years (*SD* = 3.04 years). Eighteen of the participants were females.

### Procedure

The procedure was the same as the procedure of Experiment 1, except for the use of the tongs, the inclusion of a practice task, and the absence of a second phase.

### Results

**FIGURE 3:**
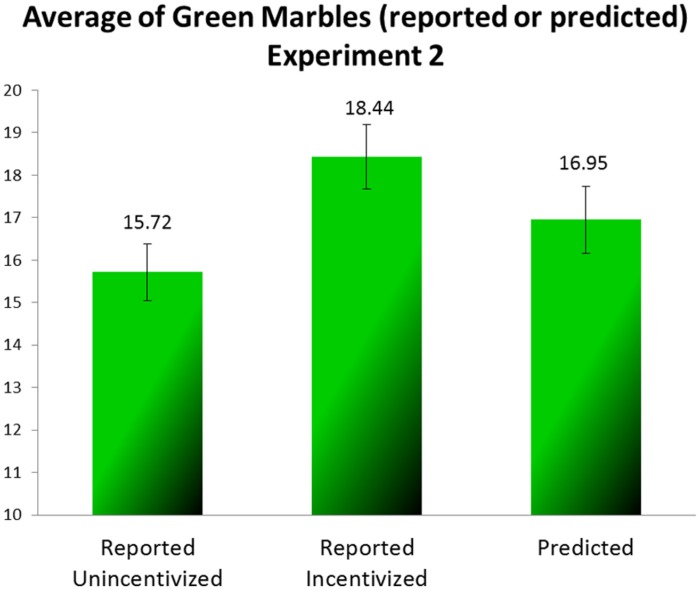
**Average of green marbles (reported or predicted) (Experiment 2)**.

The average draws (reported and predicted) of the green marbles are presented for each task in **Figure 3**. We analyzed the results across all the participants with one-way repeated measures ANOVA. We found a significant difference between the three tasks [*F*(2,76) = 9.398, *MSE* = 7.687, *p <* 0.001, $\eta_{p}^{2}$ = 0.198]. The mean reported in the first, unincentivized task was closest to the expected value of 14, the mean reported in the second, incentivized, task was much higher, and the mean in the predicting task was somewhere in between. A finer contrast analysis that compared the mean number of green marbles in the predicting task separately to the mean reported in the practice task and to that reported in the incentivized task showed the prediction to differ significantly from both [*F*(1,38) = 4.787, *MSE* = 12.340, *p* = 0.035, $\eta_{p}^{2}$ = 0.112 and *F*(1,38) = 6.050, *MSE* = 14.256, *p* = 0.019, $\eta_{p}^{2}$ = 0.137, for the practice and the incentivized reporting tasks, respectively]. We examined the differences between every two tasks with paired-sample *t*-tests. All of the differences were significant: the difference between the practice task and the incentivized reporting task [*t*(38) = 3.841, *p <* 0.001], the difference between the incentivized reporting task and the prediction task [*t*(38) = 2.46, *p* = 0.019], and the difference between the prediction task and the practice task [*t*(38) = 2.188, *p* = 0.035].

These results replicate the results of Experiment 1: apparently at least some of the participants reported more green marbles than they had really drawn, that is to say, they lied. In the prediction task the participants predicted a lower number of green marbles than they had reported, but their prediction was not as low as what they had most likely seen in the two preceding reporting tasks.

**FIGURE 4:**
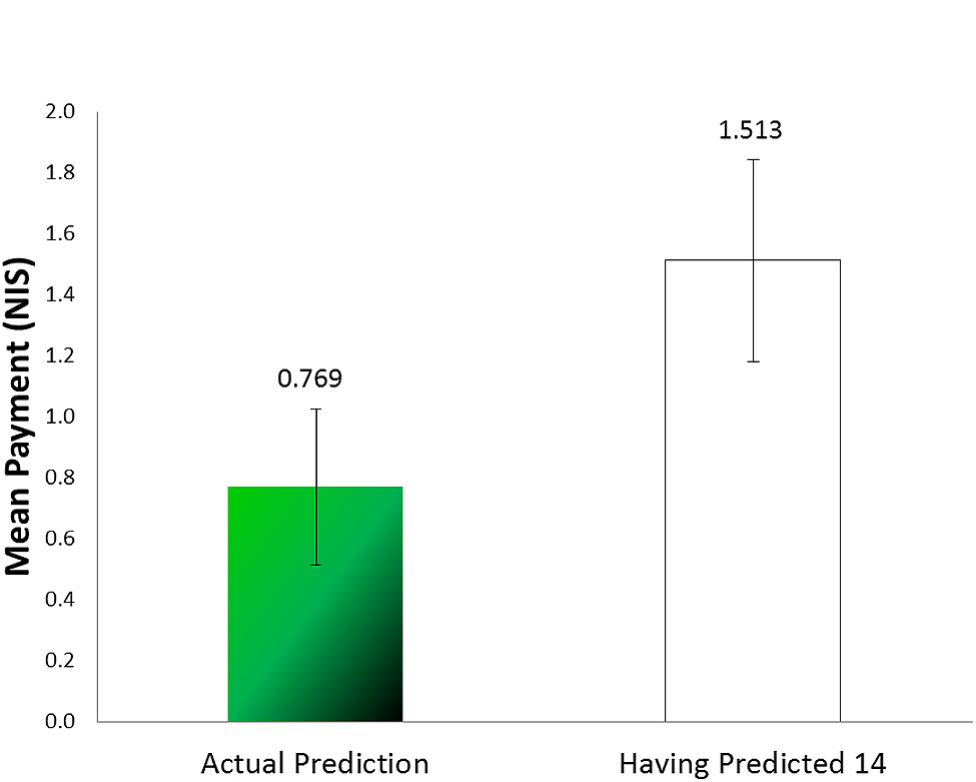
**Mean payment for prediction compared to a prediction of 14 (Experiment 2)**.

Because the number of rewarding marbles observed by each subject is unknown, it is impossible to calculate how much profit participants had foregone by being bound by their lies. For approximation we calculated the mean payment the participants would have earned had they predicted the expected value (14). In that case they would have earned 1.513 NIS (see **Figure 4**); the difference between that and what they earned is significant [*t*(38) = 1.842, *p* = 0.036, one tailed, paired sample *t*-test].

The number reported during practice also differed from the expected value (14) [*t*(38) = 2.555, *p* = 0.015]. We have no explanation for this deviation, which could have resulted by chance. In any case, it does not bear on our main finding.

In Experiment 2 we not only replicated the findings of Experiment 1, but also ruled out an alternative explanation based on anchoring. The participants sampled the urn twice, and could anchor on either of the two values reported. The up deviation of the predicted value (16.95) from both the value expected by chance (14) and the value reported and displayed in the practice task is a clear indication that participants decided to stick to their inflated reports. At the same time, the difference between the reporting that was incentivized (18.44) and the subsequent prediction indicates not only that the participants were likely aware of the true proportion of green marbles in the urn, but also that they tried to “reduce” the damage caused by sticking to their inflated reports.

## Experiment 3

Our main thesis is that behavior following a lie is affected by the lie in that the liar attempts to ensure that the act of lying is not exposed, even at a cost. Still, it could be claimed that what we regarded as the tell-tale indication of such an attempt – the large deviation of the prediction from the value expected by chance – resulted from other factors such as a failure to correctly estimate the proportion of the infrequent-color (green) marbles in the urn, self-deception (as would be predicted by the theory of self-concept maintenance Mazar et al., 2008; Chance et al., 2011), or still, by some anchoring. To test these alternative explanations we created in Experiment 3 a *non-binding* situation: not only could participants exaggerate their “report” of the number of rewarding marbles drawn with impunity, but also no one could tell how many rewarding marbles they reported. Thus, it would be impossible to find out if their prediction differed from their report, which would have implicated them as liars. We reasoned that under such conditions people would not be bound to their lies. On the other hand, if inflated predictions were the results of failures of estimation, self-deception, or anchoring they would remain higher than expected by chance, as was observed in the previous two experiments.

### Method

This experiment was similar to Experiment 2 but with some changes: only in the practice stage did participants report into a computer. In the second stage, to allow participants to inflate their “reports,” and still not be connected to that “report” (so that no one could tell if their prediction deviated from it), they had to count the number of draws and the number of green marbles for themselves. That way we could not tell, for any individual participant, how many green marbles she claimed to have drawn. Participants could use pen and paper but it was not obligatory. It was emphasized in the instructions that even if the participants were to use such aids, they would keep them and the experimenter would have no access to them.

At the end of the drawing the participant was presented with a large number of unmarked envelopes, each containing 40 0.50-shekel coins, and was asked to select one of the envelopes at random and take out as many coins as there were green marbles in the sample previously drawn, then place the unmarked envelope, with the remaining, unclaimed coins, in a large box. Because several participants performed the experiment at the same time we could not tell, for any individual participant, how many green marbles she had claimed to draw. At the same time, we could easily find out how many coins, on average, the participants had taken. Following that stage each participant engaged in the prediction task, in a different room.

### Participants

Forty three students of the Hebrew University of Jerusalem were recruited for the experiment. The average age was 24.35 years (*SD* = 2.43 years). Nineteen of the participants were females.

### Procedure

After the experimenter made sure that the participants could tell the difference between the marbles’ different colors they entered the lab in groups of 4–8 participants in each session. Each participant worked alone at her own pace in a different cubicle. It was made clear to the participants that the experimenter could not see what they were doing. The participants got the materials – urn, tongs, instructions, pen, and a sheet of paper – and began the practice task, reporting into the computer. When they finished doing that, they raised their hand and the experimenter came in and gave them the written instructions for the second task. He made sure that they understood the instructions and went into the other room. The participants repeated the procedure, this time without the computer.

Participants were then instructed to select one of the unmarked envelopes and to take as many coins out of the 40 as the number of green marbles they had previously drawn from the urn. They then sealed the envelope and put it in the box.

After the participants put the envelope in the box, they took the same urn they used before and went to the next room, where they performed the prediction task as in Experiments 1 and 2.

### Results

**FIGURE 5:**
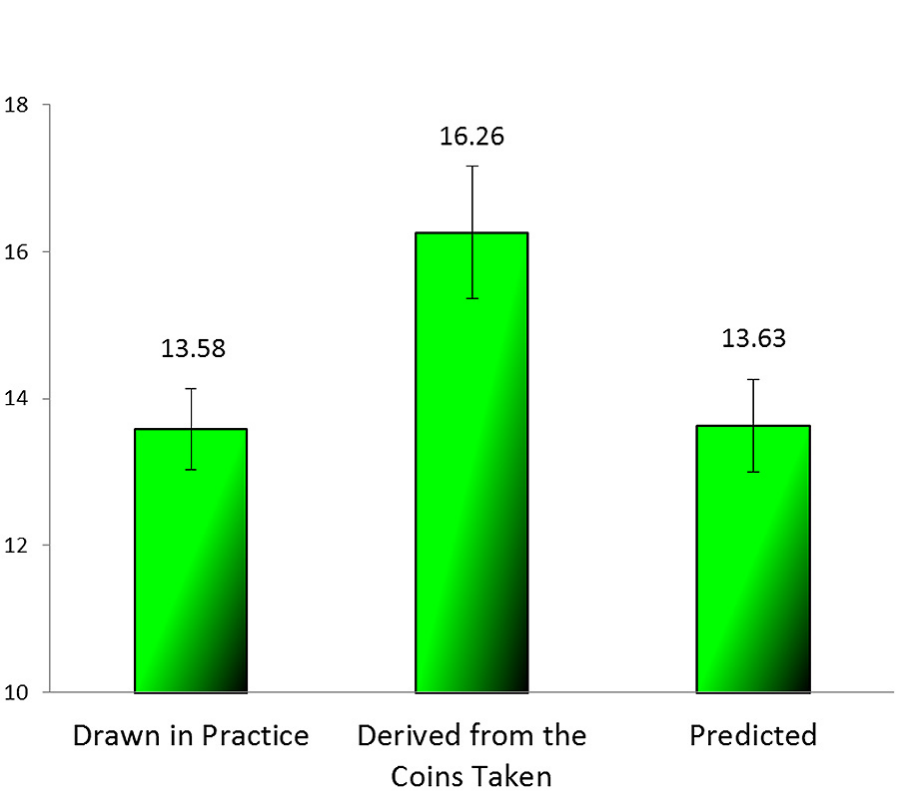
**Average of green marbles (those reported in the practice, those derived from the coins taken, and those predicted) (Experiment 3)**.

**FIGURE 6:**
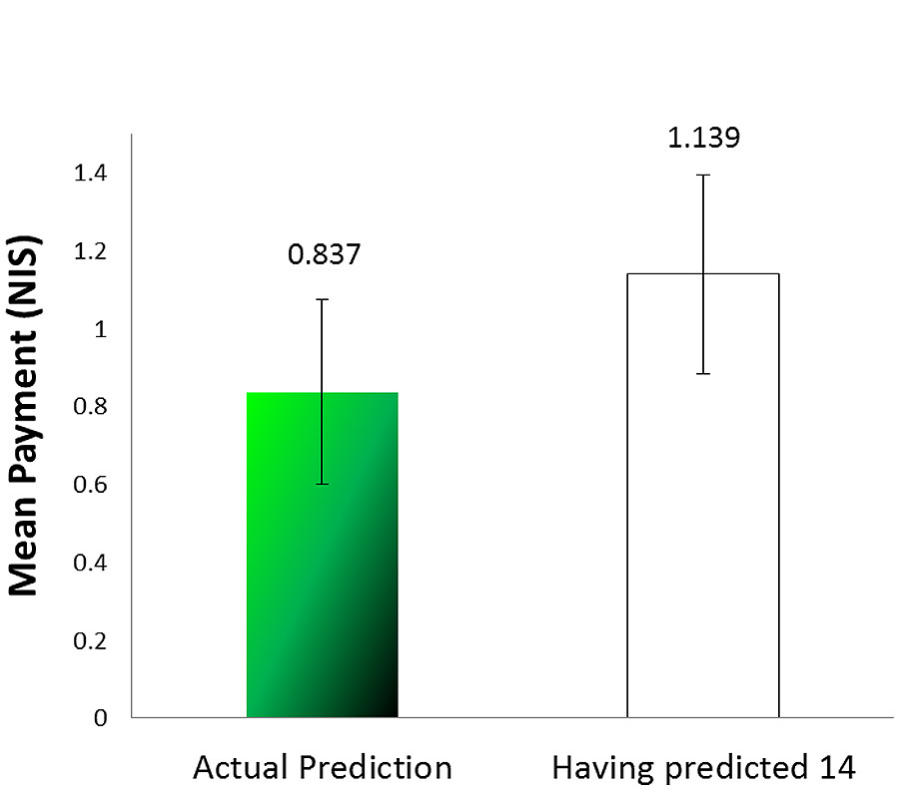
**Mean payment for prediction compared to a prediction of 14 (Experiment 3)**.

As mentioned before, the expected number of green marbles was 14. **Figure 5** presents the average number of green marbles as reported in the practice task, as derived from the number of coins removed from the envelopes, and predicted. A one-way ANOVA revealed a significant effect of stage [*F*(2,126) = 4.66, *MSE* = 21.625, *p* = 0.011, $\eta_{p}^{2}$ = 0.069). A *post hoc* comparison revealed that the mean number of coins claimed (corresponding to the values “reported” in the incentivized stage) was significantly different from both the value reported in the practice task and that predicted (*p* = 0.031, *p* = 0.035 respectively), whereas the values of the practice and the predicting task were not different from each other (*p* = 0.999). Furthermore, one sample *t*-test revealed that the average of the green marbles in the incentivized reporting task was significantly different from the expected value of 14 [*t*(42) = 2.25, *p* = 0.016], whereas the other two values were not [*t*(42) = -0.751, *p* = 0.457 for the practice task, and *t*(42) = -0.599, *p* = 0.553 for the predicting task]. The earnings in the prediction phase (Mean Payment = 0.837, *SD* = 1.557) were not significantly different from 1.139, that they would have earned had they predicted 14 [*t*(42) = -0.897, *p* = 0.375) (see **Figure 6**).

The results of this experiment clearly demonstrate that when the lie was anonymous and there was no one who could call it out, the participants were no longer bound by it. Their predictions show that given such “non-bindingness” they easily made a prediction commensurate with the actual number of marbles of the infrequent color, and maximized their profits. It should be noted that, although in this experiment we couldn’t distinguish between lying and steeling as did Mazar and Zhong (2010), given that participants were instructed to take out the number they sampled, the excess coins they took can be regarded as stealing by lying.

## General Discussion

The goal of this research was to study aspects of behavior following a lie. Specifically, we asked to what extent people would be ready to forgo a benefit in order not to imply, by a future action, that they had previously lied. We devised a sequence of two tasks, both involving the state of the same world. The first task allowed for profitable, voluntary lying behavior, in which the participants were assured, by the nature of the task that the experimenter could not tell if and when they had lied. Yet, a comparison of the overall statistical characteristics of a participant’s report with the statistical characteristics of the environment could indicate the likelihood that lying had taken place. In the subsequent unexpected task, benefits would have been higher if the true state of the world, rather than that implied by one’s previous reports, were used. As the second task was unexpected, the benefits of previously reporting the true state of the world could not be foreseen. This setup created a possible dilemma for liars, because deviation from their report in the initial task would constitute an implicit admission of having lied. The way our participants resolved the dilemma, when it existed, allowed us to assess the degree to which false reports bound the participants later on.

In Experiment 1, we have shown that people are willing to risk future profit or even forgo it altogether, in order not to get caught in a lie. The explanation we offer for such behavior is that people are “bound” to a lie they told, and are compelled by it to a certain behavior. That is, after providing the experimenter with a false report of the proportion of the profitable marbles, that person feels committed to that false representation in the sense that the person subsequently continues to predict a higher proportion than the proportion that would have most probably yielded a larger gain (but which would have been hard to justify in light of the previous report).

In Experiment 2, we have replicated these results and eliminated an alternative explanation based on anchoring, by repeating the reporting task with no incentive to lie – a task in which reporting turned out to be much closer to the value expected on statistical grounds.

In Experiment 3, we have shown that when there is no audience to the false presentation then there is no commitment to the lie. By creating a situation of a non-binding lie and showing that in that case participants felt free to act differently (and in line with what was more profitable) in the reporting and the predicting task, we have dismissed alternative explanations like an inability to correctly estimate the proportion of marbles of the infrequent color in the urn, self-deception, or anchoring.

It is important to note that the commitment to the lie does not stem from the risk of punishment, as even if inconsistent reports in the two tasks indirectly indicated that a person had lied, there was no sanctioning mechanism in our studies. Furthermore, when misrepresentation cannot be detected, as in Experiment 3, participants did not seek to be coherent with their reports.

The theory of self-concept maintenance (Mazar et al., 2008) explains well why participants did not lie “all the way” when they had an opportunity to do so. It is possible that the extra gains they made were exactly what struck a balance between monetary temptation and keeping one’s self-image intact. This theory also provides an explanation for the self-deception in performance prediction described in Chance et al. (2011). In the latter, participants may have been unaware of how much they were aided by the answers sheet, and that enabled them to deceive themselves unlike in our experiments. In the experiments reported here every lie was very prominent for the participants as they held the marble of the unprofitable color in their hand and clicked the profitable color key on the computer screen (Experiments 1 and 2) or marked it on paper (Experiment 3). It might be that failing to maintain a self-concept of honesty, people proceed to (perhaps less desirable) honest impression management. It could be that when participants in Mazar and Hawkins (2015) who inflated their performance evaluation after lying, may also have engaged in impression-management rather than in deceiving themselves.

The contrast in prediction behavior between Experiment 3 and the previous 2 experiments shows that the commitment to the lie is of impression management and not a result of self-deception.

All in all our results indicate that people feel bound by their lies and that, once having told a lie, they are willing to risk future profits in order not to be exposed as having lied.

Not all lies commit liars. Additional research should address the motivation of lying and classify the situations in which lies bind the liar. We think that people sometimes use lies as means of impression management and, as long as possible, would prefer to deceive oneself rather than admit to have lied. But when this is impossible, *looking* honest would become an important target behavior, even if costly. Impression management could be particularly strong for lies in which one embellishes reality, presenting oneself as better as or more competent than one really is. At the same time, impression management using lies could have a positive effect through binding, by becoming a commitment to the lie, a motivation and a tool for establishing and improving self-identity.

## Conflict of Interest Statement

The authors declare that the research was conducted in the absence of any commercial or financial relationships that could be construed as a potential conflict of interest.
